# Associations Between Sociodemographic Characteristics, eHealth Literacy, and Health-Promoting Lifestyle Among University Students in Taipei: Cross-Sectional Validation Study of the Chinese Version of the eHealth Literacy Scale

**DOI:** 10.2196/52314

**Published:** 2024-07-18

**Authors:** Dan-Ping Chao

**Affiliations:** 1 Department of Tourism and Leisure Management China University of Technology Taipei Taiwan

**Keywords:** college students, online health information, eHealth literacy, search literacy, usage literacy, evaluation literacy, health-promoting behavior

## Abstract

**Background:**

The popularization of the internet and rapid development of mobile devices have led to an increased inclination and opportunities to obtain health-related information online. The eHealth Literacy Scale (eHEALS), widely used for measuring eHealth literacy, assesses an individual’s ability to search, understand, appraise, and use eHealth information. However, the Chinese version of the eHEALS multiple-factor model remains to be validated, and the correlation between eHEALS and the health-promoting lifestyle profile (HPLP) among university students is rarely explored in Taiwan.

**Objective:**

This study aimed to examine the fit, validity, and reliability of the Chinese eHEALS multiple-factor model and to clarify the predictive effects of eHEALS on the HPLP among university students.

**Methods:**

University students in Taipei, the capital of Taiwan, were recruited, and 406 valid questionnaires including sociodemographic characteristics, eHEALS, and HPLP responses were collected. Confirmatory factor analysis was performed to validate the Chinese eHEALS. Independent sample *t* test, 1-way ANOVA, and multiple linear regression analyses were conducted to examine the relationship between sociodemographic variables and the HPLP. Pearson product-moment correlation and binary logistic regression analyses were performed to ascertain the predictive effects of eHEALS on the HPLP.

**Results:**

The Chinese eHEALS exhibited an optimal fit when delineated into the search, usage, and evaluation 3-factor model (comparative fit index=0.991, Tucker-Lewis index=0.984, root mean square error of approximation=0.062), and its validity and reliability were confirmed. The mean eHEALS score of university students was 3.17/4.00 (SD 0.48) points, and the score for the evaluation subscale was the lowest (mean 3.08, SD 0.56 points). Furthermore, there were significant sex, institution orientation, daily reading time, daily screen time, primary information channel, and perceived health status differences in the HPLP: male participants (*t*_404_=2.346, *P*=.02), participants attending general university (*t*_404_=2.564, *P*=.01), those reading ≥1 hour daily (*F*_2,403_=17.618, *P*<.001), those spending <3 hours on mobile devices or computers daily (*F*_2,403_=7.148, *P*<.001), those acquiring information from others (*t*_404_=3.892, *P*<.001), and those with a good perceived health status (*F*_2,403_=24.366, *P*<.001) had a significantly higher score. After adjusting for sociodemographic variables, the eHEALS score remained an independent predictor of the HPLP. Compared to students with relatively high eHEALS scores, those with relatively low eHEALS scores had a 3.37 times risk of a negative HPLP (adjusted odds ratio [OR]=3.37, 95% CI 1.49-7.61), which could explain 14.7%-24.4% of the variance (Cox-Snell *R*^2^=0.147, Nagelkerke *R*^2^=0.244, *P*=.004).

**Conclusions:**

There is room for improvement in eHealth literacy among university students in Taipei. eHEALS may be used to screen students who require HPLP improvement, thereby providing appropriate eHealth literacy training programs, particularly those targeting evaluation literacy. Additionally, the 3-factor model of the Chinese eHEALS used in this study results in more definite scale content, thus increasing the practicality and applicability of this scale in health-promoting studies.

## Introduction

### Background

In recent years, the accessibility and convenience of the internet have increased. This has allowed the public to use it more frequently for communication, education, work, or recreation. A Taiwan Network Information Center (TWNIC) survey revealed that the percentage of individuals with internet access has remained above 80% since 2015 and reached 84.3% in 2022 [1]. Notably, generation Z (aged ≤25 years) had an internet access rate of 100%, and 20.39% of the general population consistently maintained an active online status [1]. Nevertheless, there exists an opportunity for enhancing public digital literacy [1]. Recent studies have highlighted that 81% of adults in the United States possess the ability to search the internet, with 72% using online sources for health-related information [2]. The rapid and discreet nature of the internet considerably increases the public’s inclination to use the internet for accessing health-related information [3,4]. However, online health information may contain complex, inaccurate, or even misleading content, resulting in low comprehensibility and reliability [5]. Individuals may inadvertently jeopardize their well-being if they lack the ability to comprehend and critically evaluate online health information [6]. Consequently, the concept of eHealth literacy has gradually attracted attention.

Health literacy refers to the ability of an individual to engage with health information. The World Health Organization defines health literacy as “the cognitive and social skills which determine the motivation and ability of individuals to gain access to, understand and use information in ways which promote and maintain good health” [7,8]. This concept can be further divided into 3 levels, namely basic/functional, communicative/interactive, and critical [9]. Studies have indicated that individuals with high health literacy tend to effectively comprehend medical information and frequently engage in health-promoting behaviors, thereby cultivating a healthier lifestyle [10]. eHealth literacy refers to the aptitude for sourcing, comprehending, assessing, and applying health information from the internet to address health problems. Scholars have used the lily model to delineate the 6 fundamental competencies in eHealth literacy [11]. In addition to the aforementioned health literacy, these 6 competencies in eHealth literacy extend to traditional, information, scientific, media, and computer literacies [11]. eHealth literacy has been positively correlated with health literacy [12]. Studies have highlighted that eHealth literacy may potentially affect the intention and behavior of an individual to use online health information [13]. Individuals with high eHealth literacy tend to actively search and review health information online, leveraging it to enhance their health behaviors and self-manage their health care needs [14].

The eHealth Literacy Scale (eHEALS) was the first lily model–based tool developed for measuring eHealth literacy [15]. Exploratory factor analysis (EFA) confirms that eHEALS consists of a single factor with 8 questions [15]. The scale is designed to be user friendly, demonstrating strong validity, reliability, and stability [15]. In systematic reviews, eHEALS is the most commonly used tool for eHealth literacy evaluation beyond its initial publication [16]. Both the original and translated eHEALS versions are widely used across different countries and populations [16]. However, limitations exist. Studies have highlighted that eHEALS only assesses 1 dimension, rendering it difficult to effectively evaluate diverse eHealth literacy aspects [17]. Another study noted that the rapid spread of social media and mobile devices in recent years could potentially render eHEALS inadequate in completely capturing the contemporary eHealth literacy of individuals [18]. Nonetheless, although EFA is extremely useful for reducing many questions to a manageable amount, only confirmatory factor analysis (CFA) of a multiple-factor model can rigorously evaluate the one-dimensionality of the scale [19]. Recent studies using CFA have found that eHEALS is not a unidimensional concept, and the fit of the 2-factor model is better than that of the 1-factor model but still not good enough [20]. Therefore, researchers have further recommended using a 3-factor model of the original eHEALS as it has a better fit and can effectively measure an individual’s current eHealth information skills and comfort [21]. An attempt was made to divide the Chinese eHEALS into 4 factors for discussion; however, some factors only cover 1 question, and the method for dividing the factors and the model fit have not been determined [22]. In summary, as eHEALS has only 8 questions, it is more suitable to divide it into 2 or 3 factors. However, further study is required to determine the fit, validity, and reliability of the Chinese eHEALS with 2-factor or 3-factor models.

Researchers have emphasized that eHealth literacy is not a static trait but evolves in response to changes in individual circumstances, societal dynamics, and environmental factors [11]. Several studies have identified variations in eHealth literacy across different sexes, educational backgrounds, income levels, health status, degree of health concern, and frequency of health-related discussions [3,15,17,18,23,24]. eHealth literacy is considered to be positively correlated with many health behaviors adopted by an individual. Recent studies have highlighted that individuals with high eHealth literacy have positive social relationships, a balanced diet, and safe sex practice [13]. Researchers have found that people with high eHealth literacy exercise and eat breakfast regularly [18]. Studies have also proved that high eHealth literacy predicts balanced eating, regular exercise, and good sleep behaviors [17,23]. Additionally, cross-sectional and longitudinal studies have indicated that people with high eHealth literacy can successfully cultivate a health-promoting lifestyle that includes health responsibility, exercise, nutrition, self-actualization, stress management, and interpersonal support [4,24,25].

In Taiwan, adults aged 18-29 years, often called digital natives, have grown up with the internet. The TWNIC survey revealed that less than 1% of this demographic has never used the internet [1]. Notably, permanent online engagement is particularly pronounced in this population, inherently amplifying opportunities for internet-based information retrieval and usage [1]. Entering university is an important stage when adolescents transition into adulthood. Studies have shown that when presented with substantial internet-based health information encompassing both accurate and misleading content, university students may encounter challenges in accessing dependable sources and using effective evaluation methods, underscoring the need for continued strengthening of their eHealth literacy [3,18,26]. Moreover, although in good health, university students tend to exhibit risky health behaviors [27,28]. However, limited Taiwanese studies have explored the correlation between eHEALS and the health-promoting lifestyle profile (HPLP) among university students, highlighting an urgent need to ascertain and address their eHealth literacy educational requirement.

### Objective

This study aimed to evaluate the fit, validity, and reliability of Chinese eHEALS 2- and 3-factor models. Moreover, the relationship between eHealth literacy and the HPLP among university students was explored. Specifically, this study sought to uncover sociodemographic factors capable of confounding a health-promoting lifestyle among university students and to ascertain the predictive effects of eHealth literacy on adopting a health-promoting lifestyle by excluding the influence of sociodemographic confounders. Finally, the study proposed health education advice that aligns with the current trends, addressing the specific requirements of individuals with lower eHealth literacy who need prompt intervention.

## Methods

### Study Design and Participants

This cross-sectional quantitative study was conducted among university students in Taipei, the capital of Taiwan. Two rounds of testing (pretest and formal) were performed, and 2-stage sampling was used in both rounds and the subjects were not repeated. The pretest was conducted to determine the reliability of the Chinese versions of eHEALS and the HPLP in the study population and to conduct EFA to extract the 2- and 3-factor models of the Chinese eHEALS. Next, the formal test was conducted to conduct CFA to further determine the fit, validity, and reliability of the Chinese eHEALS multiple-factor models and to perform inferential statistics on the predictive effects of eHEALS on the HPLP.

In the first stage of the pretest, stratification was conducted based on the types of universities in Taipei. The Taiwan Ministry of Education has classified 24 universities in Taipei based on ownership and educational goals into 6 (25%) public general universities, 5 (21%) public vocational colleges, 8 (33%) private general universities, and 5 (21%) private vocational colleges [29]. One school was randomly selected from each stratum for testing. Subsequently, in stage 2, convenience sampling was conducted in the 4 schools. Responses from 205 subjects aged 18-22 years were collected from September to October 2020, resulting in 201 valid questionnaires being completed and returned, with an effective recovery rate of 98%.

In stage 1 of the formal test, the same method was used to divide universities in Taipei into 4 strata, and 9 (38%) of 24 schools were randomly selected from the 24 universities based on proportional stratification, including 2 (22%) public general universities, 2 (22%) public vocational colleges, 3 (33%) private general universities, and 2 (22%) private vocational colleges. Before stage 2, this study required participation from at least 384 respondents. This need was calculated using the following formula for determining the sample size [30]: where *χ*^2^, *P*, and d are known in the reference and represent the value of the chi-square for 1 degree of freedom at the desired confidence level (3.841), the population proportion (assumed to be .5, as this would provide the maximum sample size), and the degree of accuracy expressed as a proportion (0.05), respectively [30]. N represents the population size. The total number of university students in Taipei in 2021 (N=250,939) according to the Taiwan Ministry of Education [31], was substituted into in the following formula:



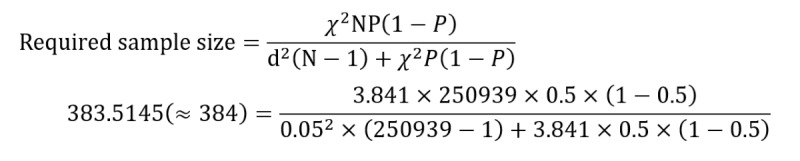



Considering the possibility of 5%-7% of the questionnaires being invalid, the number of respondents in the formal test was expected to increase to 410. Additionally, the number of respondents for each school was calculated based on the ratio of the total students in each school to those in Taipei. Subsequently, stage 2 was performed from March to April 2021, with 39-50 university students randomly selected based on their student ID numbers obtained from each school. Invitations to participate in this study were sent using the students’ school email addresses. Students who considered themselves physically and mentally stable and who accepted the invitation were included in this study. They completed the questionnaires at their respective schools at the agreed time. If the initially selected students declined to participate, respondents were drawn again as substitutes. Ultimately, 411 formal questionnaires were collected, resulting in 406 valid completed and returned questionnaires, for an effective recovery rate of 98.8%.

### Ethical Considerations

This study was reviewed and approved by the Behavioral and Social Science Research Ethics Committee of National Taiwan University (approval number 202004ES028). The ethical principles of the Declaration of Helsinki were adhered to during the entire study. Interviewers provided participants with questionnaires and explained the study’s objectives, procedures, benefits, and potential risks. The self-administered questionnaires were anonymously completed by participants in both rounds of testing after providing signed informed consent. Participants received a small gift—a pen worth New Taiwan dollar (NT $) 38 (US $1.2)—upon questionnaire completion as a token of appreciation.

### Measurements

The structured questionnaire with closed-ended items encompassed sociodemographic characteristics, eHealth literacy, and health-promoting lifestyle. Sociodemographic variables considered as potential confounders in this study included sex, institution ownership, institution orientation, living status, parental education level, religious affiliation, monthly disposable amount, daily reading time, daily screen time (on mobile devices and computers), primary information channel, and perceived health status. eHealth literacy and health-promoting lifestyle were the study’s main predictor and outcome.

The Chinese eHEALS, a translation of the original eHEALS by Norman and Skinner [15] conducted by Cheng et al [22], was used to assess eHealth literacy (Multimedia Appendix 1). As the translation process of the Chinese eHEALS was not mentioned by Cheng et al [22], 2 language teachers from the Center for General Education of the China University of Technology were invited to inspect and confirm the translation accuracy and fluency of the scale. Six experts on health science and health education reviewed the scale and found that the content validity was good (mean item content validity index=1.00, SD 0.02). This scale was initially constructed with a single factor and comprised 8 questions. A 4-point Likert scale was used for scoring in this study, ranging from 1 (strongly disagree) to 4 (strongly agree). A higher mean score reflected better self-perceived online health information skills and comfort. In binary logistic regression, mean scores for eHEALS of 1.00-3.16 and 3.17-4.00 denoted relatively low and high eHealth literacy, respectively, to facilitate subsequent explanation and application. The scale exhibited good reliability with a Cronbach α of .94. Recent studies have pointed out that the German eHEALS 2-factor model and the English eHEALS 3-factor model have a better fit than the original 1-factor model and are more meaningful [20,21]. Therefore, EFA was performed on pretest data to extract 2- and 3-factor structures from the Chinese eHEALS. The 3-factor model included search (3 questions), usage (2 questions), and evaluation (3 questions), with factor loadings ranging from 0.777-0.829, 0.753-0.833, and 0.717-0.840, respectively. The cumulative explained variance was 85.9%. The 2-factor model included search/usage (5 questions) and evaluation (3 questions), with factor loadings ranging from 0.773-0.826 and 0.758-0.853, respectively. The cumulative explained variance was 79.6%. Subsequently, CFA was performed on the formal test data to determine the fit, validity, and reliability of the 1-, 2-, and 3-factor models.

The HPLP, initially developed by Walker et al [32], was translated into Chinese by Huang and Chiou [33] and further adapted for simplification by Wei and Lu [34]. The simplified version of the Chinese HPLP was used in this study to assess a health-promoting lifestyle (Multimedia Appendix 2). The Chinese HPLP has undergone language revision and content validity review [33], is considered to be faithful to the original scale, and does not have excessive additional content [34]. The scale included 6 subscales, namely self-actualization, health responsibility, exercise, nutrition, interpersonal support, and stress management, each containing 4 items. A 5-point Likert scale was used for scoring, ranging from 1 (never) to 5 (always). A higher mean score indicated a more favorable health-promoting lifestyle. In binary logistic regression, mean scores above or equal to the middle (3/5) denoted relatively positive responses [35,36], indicating active adherence to a health-promoting lifestyle to facilitate subsequent explanation and application. The Cronbach α of the pretest data for the scale was .94, demonstrating its robust reliability.

### Statistical Analysis

CFA was conducted using SPSS AMOS 28.0 (IBM Corporation). Recent studies have shown that a model exhibits a good fit when *χ*^2^/*df*<3; the comparative fit index (CFI), Tucker-Lewis index (TLI), and relative fit index (RFI)>0.95; the goodness-of-fit index (GFI), normed fit index (NFI), and incremental fit index (IFI)>0.9; and the root mean square error of approximation (RMSEA) and standardized root mean square residual (SRMR)<0.08 [37-40]. In addition, the scale is considered to have convergent validity if the standardization factor loadings of various questions are >0.7 and the average variance extracted (AVE) of various factors are >0.5 [40,41]. The correlation coefficient of 2 factors is lower than the square root of the AVE of various factors and is considered to have discriminant validity [40]. If the Cronbach α and composite reliability (CR) of the various factors are >0.7, this indicates that the reliability is good [40,42].

In this study, SPSS 23.0 was used for other inferential statistics. An independent sample 2-tailed *t* test or 1-way ANOVA combined with Scheffé post hoc analysis was conducted to present the relationship between sociodemographic characteristics and the HPLP. Multiple linear regression was performed to examine the sociodemographic variables that may affect the HPLP. The total HPLP score was used as the dependent variable, while the 12 sociodemographic variables were transformed into 16 dummy variables to serve as independent variables. Sex (reference=female), institution ownership (reference=public), institution orientation (reference=vocational colleges), living status (reference=alone), father’s education level (reference=high school or lower), mother’s education level (reference=high school or lower), religious affiliation (reference=with), and primary information channel (reference=self-searching) were each transformed into 1 dummy variable, while monthly disposable amount (reference=NT $15,001 [US $462] or more), daily reading time (reference=less than 1 hour), daily screen time (reference=6 hours or more), and perceived health status (reference=good) were each transformed into 2 dummy variables. Stepwise regression analysis used an inclusion criterion of *P*<.05 and an exclusion criterion of *P*>.10. Tolerance>0.1 and variance inflation factor (VIF)<10 were deemed free from collinearity between independent variables. Subsequently, Pearson product-moment correlation analysis was conducted to depict the correlation between eHEALS and the HPLP. Binary logistic regression was performed to determine the predictive effects of relatively low (1.00-3.16) and relatively high (3.17-4.00) eHEALS scores on both positive (3-5) and negative (1.00-2.99) HPLPs, while accounting for relevant sociodemographic factors associated with the HPLP (ie, sex, institution orientation, daily reading time, daily screen time, primary information channel, and perceived health status). A nonsignificant Hosmer-Lemeshow test indicated a good fit for the logistic regression model. *P*<.05 was considered statistically significant.

## Results

### Confirmatory Factor Analysis of eHEALS

In this study, CFA was performed to determine the fit of the Chinese eHEALS. The 1-, 2-, and 3-factor models proposed in the study were evaluated for fit (Table 1). Results showed that the fit of the 3-factor model was significantly better than that of the other models, and the diverse indicators satisfied the recommended fit indicators (*χ*^2^/*df*=2.574, CFI=0.991, TLI=0.984, RFI=0.975, GFI=0.975, NFI=0.985, IFI=0.991, RMSEA=0.062, SRMR=0.018). Furthermore, the fit of the 3-factor model used for empirical data in this study outperformed the 3-factor model proposed by Sudbury-Riley et al [21].

Table 2 shows the factor loadings, AVE, Cronbach α, and CR of various subscales in the eHEALS 3-factor model, which all met the ideal criteria for convergent validity and reliability. In the search subscale of eHEALS, the questions had factor loadings=0.886-0.950, AVE=0.827, Cronbach α=.93, and CR=0.935. In the usage subscale, the questions had factor loadings=0.857-0.893, AVE=0.766, Cronbach α=.87, and CR=0.867. In the evaluation subscale, the questions had factor loadings=0.785-0.900, AVE=0.710, Cronbach α=.88, and CR=0.880. Table 3 shows the correlation coefficients between various eHEALS factors. The correlation coefficients of 2 factors were lower than the square roots of the AVE of various factors, which suggests that the eHEALS 3-factor model has good discriminant validity.

**Table 1:** Model fit comparison of CFA^a^ for the Chinese eHEALS^b^ (N=406).

Model	*χ*^2^ (*df*)^c^	*χ*^2^/*df*	∆*χ*^2d^	∆*df*^d^	*P* value^d^	CFI^e^	TLI^f^	RMSEA^g^	SRMR^h^
3-factor structure^i^	43.762 (17)	2.574	—^j^	—	—	0.991	0.984	0.062	0.018
2-factor structure^k^	136.094 (19)	7.163	92.332	2	<.001	0.959	0.939	0.123	0.036
1-factor structure	333.727 (20)	16.686	289.965	3	<.001	0.889	0.845	0.197	0.062
3-factor structure by Sudbury-Riley et al [21]	118.985 (17)	6.999	—	—	—	0.964	0.941	0.122	0.031

^a^CFA: confirmatory factor analysis.

^b^eHEALS: eHealth Literacy Scale.

^c^All *χ*^2^ of 4 models are statistically significant.

^d^Difference with 3-factor structure proposed in this study.

^e^CFI: comparative fit index.

^f^TLI: Tucker-Lewis index.

^g^RMSEA: root mean square error of approximation.

^h^SRMR: standardized root mean square residual.

^i^Search (3 questions), usage (2 questions), and evaluation (3 questions) factors extracted using exploratory factor analysis (EFA) of pretest data.

^j^Not applicable.

^k^Search/usage (5 questions) and evaluation (3 questions) factors extracted using EFA of pretest data.

**Table 2:** Reliability and convergent validity for the Chinese eHEALS^a^ 3-factor model (N=406).

Subscale and questions			Standardization factor loading^b^		Cronbach α		CR^c^		AVE^d^
**Search**					.93		0.935		0.827
	Q1: I know what health resources are available on the internet.	0.886		—^e^		—		—	
	Q2: I know where to find helpful health resources on the internet.	0.950		—		—		—	
	Q3: I know how to find helpful health resources on the internet.	0.890		—		—		—	
**Usage**					.87		0.867		0.766
	Q4: I know how to use the internet to answer my health questions.	0.893		—		—		—	
	Q5: I know how to use the health information I find on the internet to help me.	0.857		—		—		—	
**Evaluation**					.88		0.880		0.710
	Q6: I have the skills I need to evaluate the health resources I find on the internet.	0.785		—		—		—	
	Q7: I can tell high-quality from low-quality health resources on the internet.	0.839		—		—		—	
	Q8: I feel confident in using information from the internet to make health decisions.	0.900		—		—		—	

^a^eHEALS: eHealth Literacy Scale.

^b^All standardization factor loadings were positive and statistically significant.

^c^CR: composite reliability.

^d^AVE: average variance extracted.

^e^Not applicable.

**Table 3:** Correlation and discriminant validity for the Chinese eHEALS^a^ 3-factor model (N=406).

Factors	Search	Usage	Evaluation
Search	0.909^b^	—^c^	—
Usage	0.864	0.875^b^	—
Evaluation	0.792	0.823	0.843^b^

^a^eHEALS: eHealth Literacy Scale.

^b^Square root of average variance extracted (AVE) for each factor.

^c^Not applicable.

### Sociodemographic Characteristics

Table 4 shows the sociodemographic variables in this study. In total, 406 students were enrolled in this study. Overall, 252 (62.1%) of the 406 participants were female, 224 (55.2%) lived with family members or friends, and 269 (66.3%) did not have specific religious beliefs. Regarding institution ownership and educational goals, the sample ratio was close to the distribution ratio of various universities in Taipei. More than half of the students were enrolled in private universities (n=226, 55.7%) than in public universities. Furthermore, the ratio of students attending general universities (n=227, 55.9%) was higher than that of students attending vocational colleges. Regarding the parental education level, 237 (58.4%) of the participants had fathers with a university degree or higher, while 252 (62.1%) had mothers with a university education level or higher. Most participants had a monthly disposable amount of NT $10,000 (US $308) or less (n=182, 44.8%). Additionally, a significant proportion of the participants spent less than 1 hour reading per day (n=198, 48.8%), while the majority spent 6 hours or more on mobile devices and computers daily (n=225, 55.4%). The participants indicated that their primary information-acquiring channel was self-searching (n=361, 88.9%), with only a minority (n=45, 11.1%) relying on asking others. Notably, 253 (62.3%) of the participants reported having a good perceived health status.

**Table 4:** Sociodemographic characteristics of university students (N=406).

Characteristics		Participants, n (%)
**Sex**		
	Male	154 (37.9)
	Female	252 (62.1)
**Institution ownership**		
	Public	180 (44.3)
	Private	226 (55.7)
**Institution orientation**		
	General university	227 (55.9)
	Vocational college	179 (44.1)
**Living status**		
	With others	224 (55.2)
	Alone	182 (44.8)
**Father’s education level**		
	High school or lower	169 (41.6)
	University or higher	237 (58.4)
**Mother’s education level**		
	High school or lower	154 (37.9)
	University or higher	252 (62.1)
**Religious affiliation**		
	Without	269 (66.3)
	With	137 (33.7)
**Monthly disposable amount (NT $)^a^**		
	≤10,000 (≤US $308)	182 (44.8)
	10,001-15,000 (US $308-$462)	137 (33.8)
	≥15,001 (≥US $462)	87 (21.4)
**Daily reading time (hours)**		
	<1	198 (48.8)
	1.0-2.9	159 (39.1)
	≥3	49 (12.1)
**Daily screen time (hours)**		
	<3	44 (10.8)
	3.0-5.9	137 (33.8)
	≥6	225 (55.4)
**Primary information channel**		
	Consulting others	45 (11.1)
	Self-searching	361 (88.9)
**Perceived health status**		
	Good	253 (62.3)
	Average	134 (33.0)
	Poor	19 (4.7)

^a^An exchange rate of NT $1=US $0.03 was used.

### Current eHEALS and the HPLP

In eHEALS, the mean total score was 3.17 (SD 0.48). The score of the usage subscale was the highest (mean 3.25, SD 0.50), followed by the search subscale (mean 3.20, SD 0.52) and the evaluation subscale (mean 3.08, SD 0.56). In the HPLP, the mean total score was 3.55 (SD 0.62). The score of the interpersonal support subscale was the highest (mean 3.87, SD 0.70), followed by the self-actualization subscale (mean 3.85, SD 0.74), the stress management subscale (mean 3.74, SD 0.74), the nutrition subscale (mean 3.41, SD 0.79), and the health responsibility subscale (mean 3.26, SD 0.87), with the exercise subscale (mean 3.18, SD 0.90) being the lowest.

### Relationship Between Sociodemographic Variables and the HPLP

As shown in Table 5, the total HPLP score showed significant differences between sexes (*t*_404_=2.346, *P*=.02), institution orientation (*t*_404_=2.564, *P*=.01), daily reading time (*F*_2,403_=17.618, *P*<.001), daily screen time (*F*_2,403_=7.148, *P*<.001), primary information channel (*t*_404_=3.892, *P*<.001), and perceived health status (*F*_2,403_=24.366, *P*<.001). Specifically, the HPLP score was higher for male participants (mean 3.65, SD 0.71) than female ones (mean 3.49, SD 0.55). Participants attending general university (mean 3.62, SD 0.59) had a higher HPLP score than those attending vocational college (mean 3.46, SD 0.65). Regarding daily reading time, participants who read for 1.0-2.9 (mean 3.67, SD 0.58) and ≥3 hours (mean 3.87, SD 0.49) had higher HPLP scores than those who read for <1 hour (mean 3.38, SD 0.64). Regarding daily screen time, participants who spent <3 hours (mean 3.70, SD 0.61) had higher HPLP scores than those who spent ≥6 hours (mean 3.45, SD 0.60). Additionally, the HPLP score was higher for participants who acquired information from others (mean 3.89, SD 0.59) than those who acquired information by themselves (mean 3.51, SD 0.62). Participants with a good perceived health status (mean 3.71, SD 0.62) had higher HPLP scores than those with an average (mean 3.32, SD 0.55) or a poor (mean 3.09, SD 0.50) perceived health status.

Stepwise multiple linear regression was performed to analyze sociodemographic variables that affected the HPLP of participants (Table 6). Results showed that sex, institution orientation, daily reading time, primary information channel, and perceived health status are confounders of the overall HPLP. In particular, male participants, participants attending general university, those who read for ≥1 hour daily, those who acquired information from others, and those with a good perceived health status had a better HPLP. Collinearity was absent between the independent variables (tolerance=0.825-0.969, VIF=1.032-1.212), and the factors explained 19.8% of the variance (adjusted *R*^2^=0.198, *F*_7,398_=15.290, *P*<.001).

**Table 5:** Associations between sociodemographic characteristics and the HPLP^a^ (N=406).

Characteristics		HPLP, mean (SD)
**Sex (*P*=.02^b^)**		
	Male	3.65 (0.71)
	Female	3.49 (0.55)
**Institution orientation (*P*=.01)^b^**		
	General university	3.62 (0.59)
	Vocational college	3.46 (0.65)
**Daily reading time (*P*<.001)^c^**		
	<1 hour	3.38^d^ (0.64)
	1.0-2.9 hours	3.67^e^ (0.58)
	≥3 hours	3.87^e^ (0.49)
**Daily screen time (*P*<.001)^c^**		
	<3 hours	3.70^e^ (0.61)
	3.0-5.9 hours	3.67^d,e^ (0.64)
	≥6 hours	3.45^d^ (0.60)
**Primary information channel (*P*<.001)^b^**		
	Consulting others	3.89 (0.59)
	Self-searching	3.51 (0.62)
**Perceived health status (*P*<.001)^c^**		
	Good	3.71^e^ (0.62)
	Average	3.32^d^ (0.55)
	Poor	3.09^d^ (0.50)

^a^HPLP: health-promoting lifestyle profile.

^b^From an independent sample 2-tailed *t* test for comparing dichotomized variables.

^c^From 1-way ANOVA combined with the Scheffé post hoc test for comparing variables with more than 2 categories.

^d,e^Values with different superscript letters in variables with more than 2 categories indicate significant differences by Scheffé post hoc test.

**Table 6:** Stepwise multiple linear regression for factors associated with the HPLP^a^ (N=406).

Factors		B	β	*P* value	Tolerance	VIF^b^
**Sex**						
	Male	0.159	0.124	.007	0.958	1.044
**Institution orientation**						
	General university	0.126	0.101	.03	0.940	1.064
**Daily reading time (hours)**						
	1.0-2.9	0.249	0.195	<.001	0.905	1.104
	≥3	0.319	0.167	<.001	0.825	1.212
**Primary information channel**						
	Consulting others	0.279	0.141	.002	0.932	1.073
**Perceived health status**						
	Average	–0.345	–0.261	<.001	0.969	1.032
	Poor	–0.548	–0.186	<.001	0.967	1.034

^a^HPLP: health-promoting lifestyle profile.

^b^VIF: variance inflation factor.

### Relationship Between eHEALS and the HPLP

Pearson product-moment correlation was performed to analyze the correlation between eHEALS and the HPLP (Table 7). The overall eHEALS showed a significantly moderate positive correlation with the overall HPLP among participants (*r*=0.512, *P*<.001). Furthermore, different eHEALS dimensions showed a significantly low-to-moderate positive correlation with the various HPLP dimensions (*r*=0.291-0.522, *P*<.001).

Binary logistic regression was performed to analyze the predictive effects of the overall eHEALS and its various dimensions on the overall HPLP among participants (Table 8). After adjusting for sociodemographic variables, compared with participants with relatively high overall eHEALS scores, those with relatively low eHEALS scores had 3.37 times the risk of a negative HPLP (adjusted odds ratio [OR]=3.37, 95% CI 1.49-7.61). The model exhibited a good fit (Hosmer-Lemeshow *χ*^2^_8_=2.128, *P*=.98), could explain 14.7%-24.4% of the variance (Cox-Snell *R*^2^=0.147, Nagelkerke *R*^2^=0.244), and had an accurate classification rate of 83.3%.

**Table 7:** Correlation between eHEALS^a^ and the HPLP^b^ (N=406).

HPLP items	eHEALS									
	Overall scale		Search subscale			Usage subscale			Evaluation subscale	
	r	*P* value	r	*P* value	r		*P* value	r		*P* value
Overall	0.512	<.001	0.442	<.001	0.406		<.001	0.521		<.001
Self-actualization	0.395	<.001	0.375	<.001	0.328		<.001	0.363		<.001
Health responsibility	0.481	<.001	0.410	<.001	0.336		<.001	0.522		<.001
Exercise	0.375	<.001	0.311	<.001	0.291		<.001	0.397		<.001
Nutrition	0.411	<.001	0.359	<.001	0.326		<.001	0.416		<.001
Interpersonal support	0.348	<.001	0.299	<.001	0.306		<.001	0.338		<.001
Stress management	0.397	<.001	0.327	<.001	0.336		<.001	0.406		<.001

^a^eHEALS: eHealth Literacy Scale.

^b^HPLP: health-promoting lifestyle profile.

**Table 8:** Binary logistic regression for association of eHEALS^a^ with the HPLP^b^ (N=406).

eHEALS items			HPLP			Unadjusted		Adjusted^c^		
		Positive		Negative	OR^d^ (95% CI)		*P* value	OR (95% CI)	*P* value	Model *P* value
**Overall scale**										
	Relatively low	218		62	4.20 (1.94-9.06)		<.001	3.37 (1.49-7.61)	.004	<.001
	Relatively high (reference)	118		8	—^e^		—	—	—	—
**Search subscale**										
	Relatively low	216		61	3.77 (1.81-7.85)		<.001	3.38 (1.54-7.42)	.002	<.001
	Relatively high (reference)	120		9	—		—	—	—	—
**Usage subscale**										
	Relatively low	216		58	2.69 (1.39-5.20)		.003	2.25 (1.11-4.59)	.025	<.001
	Relatively high (reference)	120		12	—		—	—	—	—
**Evaluation subscale**										
	Relatively low	233		63	3.98 (1.76-8.98)		<.001	3.20 (1.35-7.59)	.008	<.001
	Relatively high (reference)	103		7	—		—	—	—	—

^a^eHEALS: eHealth Literacy Scale.

^b^HPLP: health-promoting lifestyle profile.

^c^Adjusted for sex, institution orientation, daily reading time, daily screen time, primary information channel, and perceived health status.

^d^OR: odds ratio.

^e^Not applicable.

Compared with participants with relatively high eHEALS search subscale scores, those with relatively low search abilities had 3.38 times the risk of a negative overall HPLP (adjusted OR=3.38, 95% CI 1.54-7.42). The model exhibited a good fit (Hosmer-Lemeshow *χ*^2^_8_=3.052, *P*=.93), could explain 14.8%-24.7% of the variance (Cox-Snell *R*^2^=0.148, Nagelkerke *R*^2^=0.247), and had an accurate classification rate of 83.3%. Compared with participants with relatively high eHEALS usage subscale scores, those with relatively low usage abilities had 2.25 times the risk of a negative HPLP (adjusted OR=2.25, 95% CI 1.11-4.59). The model exhibited a good fit (Hosmer-Lemeshow *χ*^2^_8_=10.538, *P*=.23), could explain 13.7%-22.8% of the variance (Cox-Snell *R*^2^=0.137, Nagelkerke *R*^2^=0.228), and had an accurate classification rate of 82.8%. Moreover, compared with participants with relatively high eHEALS evaluation subscale scores, those with relatively low evaluation abilities had 3.20 times the risk of a negative HPLP (adjusted OR=3.20, 95% CI 1.35-7.59). The model exhibited a good fit (Hosmer-Lemeshow *χ*^2^_8_=2.916, *P*=.94), could explain 14.3%-23.8% of the variance (Cox-Snell *R*^2^=0.143, Nagelkerke *R*^2^=0.238), and had an accurate classification rate of 83.7%.

Further analysis of the prediction results of eHEALS on various dimensions of the HPLP was conducted (Multimedia Appendix 3). Results showed that compared with participants with relatively high overall eHEALS scores, those with relatively low eHEALS scores had 2.74 times the risk of negative health responsibility (adjusted OR=2.74, 95% CI 1.55-4.84), 2.41 times the risk of negative exercise (adjusted OR=2.41, 95% CI 1.43-4.07), and 1.86 times the risk of negative nutrition (adjusted OR=1.86, 95% CI 1.07-3.22).

Compared with participants with relatively high eHEALS subscales scores, those with relatively low search, usage, and evaluation abilities, respectively, had 2.66 (adjusted OR=2.66, 95% CI 1.52-4.62), 2.00 (adjusted OR=2.00, 95% CI 1.18-3.37), and 3.01 (adjusted OR=3.01, 95% CI 1.63-5.55) times the risk of negative health responsibility; 2.02 (adjusted OR=2.02, 95% CI 1.22-3.35), 2.12 (adjusted OR=2.12, 95% CI 1.29-3.50), and 2.71 (adjusted OR=2.71, 95% CI 1.54-4.76) times the risk of negative exercise; and 2.08 (adjusted OR=2.08, 95% CI 1.12-3.86), 1.83 (adjusted OR=1.83, 95% CI 1.08-3.11), and 2.08 (adjusted OR=2.08, 95% CI 1.07-4.06) times the risk of negative nutrition. In addition, compared with participants with relatively high eHEALS evaluation subscale scores, those with relatively low evaluation abilities had 2.06 times the risk of negative stress management (adjusted OR=2.06, 95% CI 1.01-4.22).

## Discussion

### Principal Findings

#### Comparison of the Chinese eHEALS 3-Factor Model With Previous Studies

Norman and Skinner [15] developed eHEALS and highlighted that men’s eHEALS scores are significantly higher than those of women, which could be used as an a priori hypothesis. Similar results were obtained by using the Chinese eHEALS in this study; in other words, significant differences were observed in eHEALS scores between sexes (*t*_404_=2.708, *P*=.007), with males having higher scores (mean 3.25, SD 0.51) than females (mean 3.12, SD 0.46). This shows that the Chinese eHEALS has known-groups validity. Moreover, in this study, the original 8 eHEALS questions were classified into 3 factors, namely search (questions 1-3), usage (questions 4 and 5), and evaluation (questions 6-8). Compared with the initial 1-factor model [15], CFA showed that the 3-factor model exhibits a better fit and good validity and reliability. The findings were similar to those of a recent study on the Chinese eHEALS multifactorial model [22]; however, this study showed more robust evidence of fit, validity, and reliability. In contrast to Sudbury-Riley et al [21], who used a 3-factor eHEALS model and defined question 3 as “I know how to find helpful health resources and information on the internet” and questions 4 and 5 as the ability to acquire and use health resources and information, this study defined questions 1-3 as the ability to search for health resources on the internet and questions 4 and 5 as the ability to use online health information. Results revealed that differences in the delineation of questions lead to variations in the model fit. Notably, empirical data showed that the fit of the 3-factor model in this study is superior to that of Sudbury-Riley et al’s [21] model. This can be attributed to 2 potential explanations. First, EFA was performed in the pretest to delineate the 3 factors, which differed from Sudbury-Riley et al’s [21] method, who carefully reviewed and partitioned the factors based on social cognitive and self-efficacy theories. Second, minor differences in participants’ perceptions of the translated scale may have contributed to these disparities [43]. In the English eHEALS, questions 3-5 start with “I know how to,” which may have caused participants to perceive them as belonging to the same factor [21]. In the Chinese eHEALS, participants tended to consider questions 1-3 as search factors due to words such as “what,” “where,” and “find,” while the word “use” in questions 4 and 5 led participants to classify it as a usage factor. Nonetheless, the 3-factor model used in this study complies with the foundational theories of the eHEALS lily model (ie, social cognitive theory and self-efficacy theory) [11,21]. This model may be more suitable for regions where the Chinese eHEALS is used in eHealth literacy studies.

#### eHealth Literacy Level

In this study, the overall eHEALS score of the university students was moderate or higher, and the search and usage dimensions had higher scores. In contrast, the evaluation dimension had a lower score. This reveals that students perceive themselves to have good search and usage capabilities of eHealth information; however, they possess low confidence in evaluating such information and using it for decision-making. In recent studies, the mean scores for eHEALS questions 6-8 were lower than those for questions 1-3 and questions 4 and 5 [3,21,44,45], similar to scores obtained in this study. A Taiwanese study used a self-formulated scale to evaluate the eHealth literacy of university students and divided the questions into functional, interactive, and critical literacies [17]. Interactive literacy encompasses the ability to select, comprehend, and use online health information, which was similar to the search and usage dimensions in this study. Critical literacy refers to the ability to analyze, criticize, and respond to online health information, which was similar to the evaluation dimension in this study. The score for critical literacy was visibly lower than that for interactive literacy in the previous study [17], which was similar to this study. Researchers found that although most university students mentioned that they can understand the general idea of online health information, they have a vague understanding of the jargon, foreign languages, and data [6]. In addition, some university students lack confidence in the quality of online health information and express difficulty in determining the quality of such information [3]. Therefore, in the contemporary landscape characterized by the unlimited accumulation and dissemination of internet-based health information of uncertain veracity, imparting fundamental health knowledge to Taiwanese university students is imperative. This includes fostering a sense of caution toward eHealth information among students and equipping them with the ability to critically assess and validate uncertainties.

#### Association Between Sociodemographic Variables and the HPLP

The overall HPLP of university students in this study was moderate or higher, wherein interpersonal support and self-actualization scores were the highest, while nutrition, exercise, and health responsibility scores were the lowest, similar to those of the most recent studies [46-49]. Among sociodemographic variables, stepwise multiple linear regression showed that female students, students attending vocational colleges, those with a daily reading time of <1 hour, those who acquired information by themselves, and those with an average or a poor perceived health status were confounders of a poor overall HPLP. This was consistent with the significant differences in the overall HPLP in these sociodemographic variables. Recent studies have found that sex affects the HPLP and health behaviors, such as exercise and sleep [17,48,49]. The frequency of discussions of health problems with others has been highlighted to positively affect the dietary behavior of university students [23]. Individuals with a good perceived health status or great concern for health have a better HPLP and show several health behaviors, such as eating, exercise, and sleep [17,23,24,47,49].

In addition, this study found that a daily reading time of ≥1 hour is a confounder of a good HPLP among university students. This may be because information in books, newspapers, and magazines usually undergoes review and proofreading, and reading more accurate and reliable hardcopy information may lead to a tendency to adopt a positive lifestyle profile. Some studies have highlighted that reading hardcopy materials can promote better comprehension results than reading from screens [50,51]. However, the increased screen time on digital media today has greatly decreased the reading time in print. In this study, only 208 (51.2%) of 406 university students read for ≥1 hour per day, but 362 (89.2%) spent ≥3 hours on mobile devices or computers daily. Recent studies have shown that newspapers and magazines are the media that Taiwanese university students spend the least time on, far below the time spent on mobile devices and computers [22]. Furthermore, mobile devices and computers have diverse online functions. The TWNIC survey found that the most commonly used internet functions among generation Z are real-time messaging, social networks, free videos, online news, online games, and ecommerce; however, online learning is not their priority [1]. In the ANOVA in this study, the HPLP score of participants with a daily screen time of ≥6 hours was significantly low. However, multiple linear regression excluded the daily screen time from the HPLP confounders. It is believed that frequent usage of mobile devices and computers by university students consumes the time spent on reading. Therefore, the effect of screen time in the multiple linear regression may be explained by the reading time factor. In the binary logistic regression in this study, daily screen time was still considered a confounder of HPLP scores.

#### Association of eHEALS With the HPLP

After adjusting for sociodemographic factors that may affect the HPLP, this study revealed that eHEALS consistently and significantly affects the HPLP of university students. Compared with students with relatively high overall eHEALS scores, those with relatively low eHEALS scores had a higher probability of a negative overall HPLP, similar to the results of studies in other regions [4,25]. Other researchers have used their own created scales to measure eHealth literacy and proved that it predicts multiple HPLP dimensions in university students [24]. Many studies have found that eHealth literacy has positive effects on exercise, diet, and sleep behaviors [13,17,18,23], or even safe sex practice [13] and COVID-19 prevention [46] among university students. This study found that among the various HPLP dimensions, compared with a relatively high overall eHEALS score, a relatively low eHEALS score is associated with negative health responsibility, exercise, and nutrition. University students in this study had low HPLP health responsibility, exercise, and nutrition scores—dimensions that require improvements. At the same time, many recent studies have found that these health behaviors are poor in university students [46-49]. Additionally, among the 3 eHEALS dimensions in this study, compared with participants with relatively high search, usage, and evaluation literacies, those with relatively low scores had a higher probability of a negative overall HPLP and its health responsibility, exercise, and nutrition dimensions, similar to the overall eHEALS results. Regarding evaluation literacy, this study found that in addition to predicting the negative health responsibility, exercise, and nutrition dimensions of the HPLP, a relatively low eHEALS evaluation score can also reflect poor stress management. This result is similar to that of a recent study indicating that critical eHealth literacy can predict more HPLP dimensions [24].

### Limitations and Strengths

This study has certain limitations, which can provide a reference for future studies. First, this study included only university students without major diseases from the capital of Taiwan, and the results can only be generalized to the eHealth literacy and health-promoting lifestyle of this population. It is recommended that future studies extend to other regions in Taiwan or university students with other health statuses. Second, some variables may be related to eHealth literacy and a health-promoting lifestyle, such as majors and health risk behaviors, and it is recommended that future studies expand to include these variables. In addition, although participants were advised that the entire process was anonymized, the self-administered questionnaire may have caused their answers to be exposed to memory recall errors, environmental effects, and social desirability bias. Lastly, a cross-sectional study design was used in this study, and the causal relationship between eHealth literacy and a health-promoting lifestyle, as well as changes in these 2 factors with time, could not be confirmed. Hence, further repeated-measures or longitudinal studies are required for clarification.

Nonetheless, this study confirmed the feasibility of using the Chinese version of the eHEALS 3-factor model to examine eHealth literacy and highlighted that eHealth literacy affects and predicts the HPLP in university students. In the contemporary world where internet use is widespread and portable mobile devices are rapidly advancing, using the internet, mobile phones, tablets, or computers as aids in daily life has become an unstoppable trend. If university students can cultivate the online learning habit early on and establish the concept of consulting to acquire information and reading in print, actively nurturing their skills to search, use, and access internet-based health information, it will undoubtedly positively impact their health-promoting behavior and lifestyle.

### Conclusion

This study is the first to validate the Chinese eHEALS 3-factor model, encompassing search, usage, and evaluation dimensions. Notably, eHEALS is the first eHealth literacy measurement tool to be developed and is the most widely used. This 3-factor model results in more definite eHEALS content and undoubtedly increases the practicality and applicability of the scale to satisfy the eHealth literacy evaluation needs of health promoting–related studies, particularly in Chinese-speaking regions.

Higher education represents the most significant and final opportunity for behavioral development and learning in young people. Behavioral health during this period impacts lifetime health outcomes. This study found that alongside specific sociodemographic characteristics, the overall eHEALS and its dimensions are independent predictors of the HPLP. Compared to university students with relatively high overall eHEALS and various dimension scores, those with relatively low scores had a negative overall HPLP and HPLP health responsibility, exercise, and nutrition. University students with relatively low eHEALS evaluation scores compared to those with relatively high evaluation scores also had negative stress management. These findings can be used to screen university students who require HPLP improvement so that health education suitable for their needs can be provided.

In addition, there is room for improving overall eHEALS scores among university students, with particular attention to improving evaluation literacy. It is recommended that the centers for general education, digital learning, and health of the universities and colleges in Taipei, as well as targeting populations with relatively low eHealth literacy (eHEALS score<3.17), be integrated to provide appropriate health education and programs. Courses should be conducted to educate students on identifying objective, credible, and understandable online health information platforms, while cultivating vigilance and critical judgment in evaluating eHealth information. Additionally, fostering a supportive and user-friendly online health information environment is essential. It is recommended that universities and colleges further establish good campus eHealth literacy learning and support channels. For example, good health information online platforms could be recommended on school websites, and in-person or virtual health information consultation could be applied within schools. These measures would collectively contribute to improving university students’ eHealth literacy, thereby encouraging their adoption of health-promoting lifestyles.

## Appendix

Multimedia Appendix 1 The Chinese version of eHEALS. eHEALS: eHealth Literacy Scale.

Multimedia Appendix 2 The simplified version of Chinese HPLP. HPLP: health-promoting lifestyle profile.

Multimedia Appendix 3 Binary logistic regression for association of eHEALS with the HPLP dimensions (N=406). eHEALS: eHealth Literacy Scale; HPLP: health-promoting lifestyle profile.

## Abbreviations

AVE: average variance extracted
CFA: confirmatory factor analysis
CFI: comparative fit index
CR: composite reliability
EFA: exploratory factor analysis
eHEALS: eHealth Literacy Scale
GFI: goodness-of-fit index
HPLP: health-promoting lifestyle profile
IFI: incremental fit index
NFI: normed fit index
OR: odds ratio
RFI: relative fit index
RMSEA: root mean square error of approximation
SRMR: standardized root mean square residual
TLI: Tucker-Lewis index
TWNIC: Taiwan Network Information Center
VIF: variance inflation factor

## Data Availability

The data sets generated and analyzed during this study are not publicly available but are available from the corresponding author upon reasonable request.

## References

[1] (2022). 2022 Taiwan internet report. Taiwan Network Information Center.

[2] Fox S, Duggan M (2013). Health online 2013. Pew Research Center.

[3] Dashti S, Peyman N, Tajfard M, Esmaeeli H (2017). E-health literacy of medical and health sciences university students in Mashhad, Iran in 2016: a pilot study. Electron Physician.

[4] Yoğurtcu H, Ozturk Haney M (2022). The relationship between e-health literacy and health-promoting behaviors of Turkish hospital nurses. Glob Health Promot.

[5] Eysenbach G, Köhler C (2002). How do consumers search for and appraise health information on the world wide web? Qualitative study using focus groups, usability tests, and in-depth interviews. BMJ.

[6] Hsu WC, Chen SF, Ho CJ (2011). Experience of using web health information among college students: an analysis from the health literacy perspective. J Health Promot Health Educ.

[7] (1998). Health promotion glossary. World Health Organization.

[8] (2018). A practical guidebook for health literate organization. Ministry of Health and Welfare, Taiwan.

[9] Nutbeam D (2000). Health literacy as a public health goal: a challenge for contemporary health education and communication strategies into the 21st century. Health Promot Int.

[10] Yokokawa H, Yuasa M, Sanada H, Hisaoka T, Fukuda H (2015). Age- and sex-specific impact of health literacy on healthy lifestyle characteristics among Japanese residents in a rural community. Health.

[11] Norman CD, Skinner HA (2006). eHealth literacy: essential skills for consumer health in a networked world. J Med Internet Res.

[12] Nakayama K, Osaka W, Togari T, Ishikawa H, Yonekura Y, Sekido A, Matsumoto M (2015). Comprehensive health literacy in Japan is lower than in Europe: a validated Japanese-language assessment of health literacy. BMC Public Health.

[13] Britt RK, Collins WB, Wilson K, Linnemeier G, Englebert AM (2017). eHealth literacy and health behaviors affecting modern college students: a pilot study of issues identified by the American College Health Association. J Med Internet Res.

[14] Neter E, Brainin E (2012). eHealth literacy: extending the digital divide to the realm of health information. J Med Internet Res.

[15] Norman CD, Skinner HA (2006). eHEALS: The eHealth Literacy Scale. J Med Internet Res.

[16] Karnoe A, Kayser L (2015). How is eHealth literacy measured and what do the measurements tell us? A systematic review. Knowl Manag E-Learn: Int J.

[17] Chiang CH, Yang SC, Hsu WC (2015). Development and validation of the e-health literacy scale and investigation of the relationships between e-health literacy and healthy behavior among undergraduate students in Taiwan. Formosa J Mental Health.

[18] Tsukahara S, Yamaguchi S, Igarashi F, Uruma R, Ikuina N, Iwakura K, Koizumi K, Sato Y (2020). Association of eHealth literacy with lifestyle behaviors in university students: questionnaire-based cross-sectional study. J Med Internet Res.

[19] Gerbing DW, Anderson JC (1988). An updated paradigm for scale development incorporating unidimensionality and its assessment. J Mark Res.

[20] Soellner R, Huber S, Reder M (2014). The concept of eHealth literacy and its measurement. J Media Psychol.

[21] Sudbury-Riley L, FitzPatrick M, Schulz PJ (2017). Exploring the measurement properties of the eHealth Literacy Scale (eHEALS) among baby boomers: a multinational test of measurement invariance. J Med Internet Res.

[22] Cheng SY, Chang FC, Li JM (2014). eHealth literacy and related factors among junior high school students in Taipei City. J Health Promot Health Educ.

[23] Hsu W, Chiang C, Yang S (2014). The effect of individual factors on health behaviors among college students: the mediating effects of eHealth literacy. J Med Internet Res.

[24] Yang SC, Luo YF, Chiang CH (2017). The associations among individual factors, eHealth literacy, and health-promoting lifestyles among college students. J Med Internet Res.

[25] Li S, Cui G, Zhou F, Liu S, Guo Y, Yin Y, Xu H (2022). The longitudinal relationship between eHealth literacy, health-promoting lifestyles, and health-related quality of life among college students: a cross-lagged analysis. Front Public Health.

[26] Stellefson M, Hanik B, Chaney B, Chaney D, Tennant B, Chavarria EA (2011). eHealth literacy among college students: a systematic review with implications for eHealth education. J Med Internet Res.

[27] Jang HJ (2016). Comparative study of health promoting lifestyle and subjective happiness on nursing students and non-nursing students. Adv Sci Technol Lett.

[28] Musaiger AO, Awadhalla MS, Al-Mannai M, AlSawad M, Asokan GV (2017). Dietary habits and sedentary behaviors among health science university students in Bahrain. Int J Adolesc Med Health.

[29] (2020). List of colleges and universities in 2020. Ministry of Education, Taiwan.

[30] Krejcie RV, Morgan DW (1970). Determining sample size for research activities. Educ Psychol Measur.

[31] (2020). Number of students in schools at all levels - by county and city. Ministry of Education, Taiwan.

[32] Walker SN, Sechrist KR, Pender NJ (1987). The health-promoting lifestyle profile: development and psychometric characteristics. Nurs Res.

[33] Huang YH, Chiou CJ (1997). Predictors contributing to health-promoting lifestyles among college students in Kaohsiung area. Chin J Public Health.

[34] Wei MH, Lu CM (2005). Development of the short-form Chinese health-promoting lifestyle profile. J Health Educ.

[35] Al-Kandari F, Vidal VL, Thomas D (2008). Health-promoting lifestyle and body mass index among College of Nursing students in Kuwait: a correlational study. Nurs Health Sci.

[36] Lolokote S, Hidru TH, Li X (2017). Do socio-cultural factors influence college students' self-rated health status and health-promoting lifestyles? A cross-sectional multicenter study in Dalian, China. BMC Public Health.

[37] Bentler PM, Bonett DG (1980). Significance tests and goodness of fit in the analysis of covariance structures. Psychol Bull.

[38] Hu LT, Bentler PM (1999). Cutoff criteria for fit indexes in covariance structure analysis: conventional criteria versus new alternatives. Struct Equ Model.

[39] McDonald RP, Ho MR (2002). Principles and practice in reporting structural equation analyses. Psychol Methods.

[40] Hair JF, Black WC, Babin BJ, Anderson RE (2009). Multivariate Data Analysis: A Global Perspective. 7th Edition.

[41] Ping RA (2004). On assuring valid measures for theoretical models using survey data. J Bus Res.

[42] Nunnally JC (1978). Psychometric Theory. 2nd Edition.

[43] Rode N (2005). Translation of measurement instruments and their reliability: an example of job-related affective well-being scale. Metodološki zvezki.

[44] Chung S, Park BK, Nahm ES (2018). The Korean eHealth Literacy Scale (K-eHEALS): reliability and validity testing in younger adults recruited online. J Med Internet Res.

[45] Del Giudice P, Bravo G, Poletto M, De Odorico A, Conte A, Brunelli L, Arnoldo L, Brusaferro S (2018). Correlation between eHealth literacy and health literacy using the eHealth Literacy Scale and real-life experiences in the health sector as a proxy measure of functional health literacy: cross-sectional web-based survey. J Med Internet Res.

[46] Kim KA, Hyun MS, De Gagne JC, Ahn JA (2023). A cross-sectional study of nursing students' eHealth literacy and COVID-19 preventive behaviours. Nurs Open.

[47] Chu-Ko F, Chong ML, Chung CJ, Chang CC, Liu HY, Huang LC (2021). Exploring the factors related to adolescent health literacy, health-promoting lifestyle profile, and health status. BMC Public Health.

[48] Núñez-Rocha GM, López-Botello CK, Salinas-Martínez AM, Arroyo-Acevedo HV, Martínez-Villarreal RT, Ávila-Ortiz MN (2020). Lifestyle, quality of life, and health promotion needs in Mexican university students: important differences by sex and academic discipline. IJERPH.

[49] Chao DP (2023). Health-promoting lifestyle and its predictors among health-related and non-health-related university students in Taiwan: a cross-sectional quantitative study. BMC Public Health.

[50] Mangen A, Walgermo BR, Brønnick K (2013). Reading linear texts on paper versus computer screen: effects on reading comprehension. Int J Educ Res.

[51] Chen G, Cheng W, Chang TW, Zheng X, Huang R (2014). A comparison of reading comprehension across paper, computer screens, and tablets: does tablet familiarity matter?. J Comput Educ.

