# Supplementary material for: Associations Between Sociodemographic Characteristics, eHealth Literacy, and Health-Promoting Lifestyle Among University Students in Taipei: Cross-Sectional Validation Study of the Chinese Version of the eHealth Literacy Scale
Source: J Med Internet Res. 2024 Jul 18;26:e52314. doi: 10.2196/52314 (PMC11294764; doi:10.2196/52314)
Supplement: Multimedia Appendix 1 [file jmir_v26i1e52314_app1.docx]

**Multimedia Appendix 1.** The Chinese version of eHEALS^a^.

| Questions | Original eHEALS  by Norman and Skinner [15] | Chinese^b^ eHEALS  by Cheng et al [22] |
| --- | --- | --- |
| 1 | I know what health resources are available on the Internet | 我知道在網路上有哪些健康資源可以使用 |
| 2 | I know where to find helpful health resources on the Internet | 我知道在網路上的哪裡能找到有幫助的健康資源 |
| 3 | I know how to find helpful health resources on the Internet | 我知道如何在網路上找到有幫助的健康資源 |
| 4 | I know how to use the Internet to answer my health questions | 我知道如何運用網路解答健康的問題 |
| 5 | I know how to use the health information I find on the Internet to help me | 我知道如何運用網路上的健康資訊來幫助我 |
| 6 | I have the skills I need to evaluate the health resources I find on the Internet | 我能評價網路上的健康資源 |
| 7 | I can tell high quality from low quality health resources on the Internet | 我能分辨網路上健康資源品質的優劣 |
| 8 | I feel confident in using information from the Internet to make health decisions | 我有信心可以運用網路上的資訊做出健康的決定 |

^a^eHEALS: eHealth Literacy Scale.

^b^A four-point Likert scale (i.e., 1=非常不同意 [strongly disagree], 2=不同意 [disagree], 3=同意 [agree], 4=非常同意 [strongly agree]) was used for scoring in this study.
