# Supplementary material for: Associations Between Sociodemographic Characteristics, eHealth Literacy, and Health-Promoting Lifestyle Among University Students in Taipei: Cross-Sectional Validation Study of the Chinese Version of the eHealth Literacy Scale
Source: J Med Internet Res. 2024 Jul 18;26:e52314. doi: 10.2196/52314 (PMC11294764; doi:10.2196/52314)
Supplement: Multimedia Appendix 2 [file jmir_v26i1e52314_app2.docx]

**Multimedia Appendix 2.** The simplified version of Chinese HPLP^a^.

| Questions | Part of original HPLP  by Walker et al [32] | Simplified version of Chinese^b^ HPLP by Wei and Lu [34] |
| --- | --- | --- |
| 1 | Work toward long-term goals in my life | 朝生命中長遠的目標努力 |
| 2 | Look forward to the future | 對未來充滿期望 |
| 3 | Find each day interesting and challenging | 發覺每天都是充滿樂趣及挑戰的 |
| 4 | Believe that my life has purpose | 確信我的生命是有目的的 |
| 5 | Discuss my health care concerns with qualified professionals | 與合格的專業人員討論關於自己健康保健方面的事情 |
| 6 | Have my blood pressure checked and know what it is | 測量血壓，並知道自己的血壓 |
| 7 | Seek information from health professionals about how to take good care of myself | 向健康專業人員詢問如何好好照顧自己 |
| 8 | Observe my body at least monthly for physical changes/danger signs | 每個月至少一次觀察自己的身體有無異狀或病徵 |
| 9 | Perform stretching exercises at least 3 times per week | 每週做伸展運動至少三次 |
| 10 | Participate in supervised exercise programs or activities | 參加有人指導的運動課程或活動 |
| 11 | Check my pulse rate when exercising | 運動時測量自己的脈搏 |
| 12 | Engage in recreational physical activities (such as walking, swimming, soccer, bicycling) | 從事休閒性的體能活動（如散步、游泳、足球、騎腳踏車） |
| 13 | Choose foods without preservatives or other additives | 選用不含防腐劑或其他添加劑的食物 |
| 14 | Eat 3 regular meals a day | 每日三餐規律 |
| 15 | Include roughage/fiber (whole grains, raw fruits, raw vegetables) in my diet | 食用富含纖維質的食物（如全穀類、天然的水果、蔬菜） |
| 16 | Plan or select meals to include the basic 4 food groups each day | 每日吃含有五大類營養素的食物 |
| 17 | Maintain meaningful and fulfilling interpersonal relationships | 維持有意義的人際關係（指深層的，非泛泛之交） |
| 18 | Spend time with close friends | 花時間與親密的朋友相處 |
| 19 | Find it easy to express concern, love and warmth to others | 對他人表達關懷、愛及溫暖 |
| 20 | Touch and am touched by people I care about | 和我關心的人相互有身體的碰觸 |
| 21 | Consciously relax muscles before sleep | 睡覺前使自己全身肌肉放鬆 |
| 22 | Concentrate on pleasant thoughts at bedtime | 就寢時，讓自己想些愉快的事物 |
| 23 | Find constructive ways to express my feelings | 以建設性的方式來表達自己的感受（指非批評，非漫罵性，能提出具體建議） |
| 24 | Use specific methods to control my stress | 採用某些方法來減輕自己所面臨的壓力 |

^a^HPLP: health-promoting lifestyle profile.

^b^A five-point Likert scale (i.e., 1=從來沒有 [never: 0-1 time out of ten], 2=極少如此 [rarely: 2-3 times out of ten], 3=偶爾如此 [sometimes: 4-6 times out of ten], 4=時常如此 [often: 7-8 times out of ten], 5=總是如此 [always: 9-10 times out of ten]) was used for scoring in this study. The number marked for each answer referred to the frequency that the subjects thought they would adopt the action out of ten times in the situation mentioned in each item.
