# Supplementary material for: Associations Between Sociodemographic Characteristics, eHealth Literacy, and Health-Promoting Lifestyle Among University Students in Taipei: Cross-Sectional Validation Study of the Chinese Version of the eHealth Literacy Scale
Source: J Med Internet Res. 2024 Jul 18;26:e52314. doi: 10.2196/52314 (PMC11294764; doi:10.2196/52314)
Supplement: Multimedia Appendix 3 [file jmir_v26i1e52314_app3.docx]

**Multimedia Appendix 3.** Binary logistic regression for association of eHEALS^a^ with the HPLP^b^ dimensions (N=406).

| eHEALS items | | HPLP subscales (Negative/Positive) | | | | | | | | | | | |
| --- | --- | --- | --- | --- | --- | --- | --- | --- | --- | --- | --- | --- | --- |
|  |  | SA^c^ | | HR^d^ | | Exercise | | Nutrition | | IS^e^ | | SM^f^ | |
|  |  | Adjusted^g^  OR^h^  (95% CI) | *P* value | Adjusted  OR  (95% CI) | *P* value | Adjusted  OR  (95% CI) | *P* value | Adjusted  OR  (95% CI) | *P* value | Adjusted  OR  (95% CI) | *P* value | Adjusted  OR  (95% CI) | *P* value |
|  |  |  |  |  |  |  |  |  |  |  |  |  |  |
| **Overall scale** | | | | | | | | | | | | | |
|  | Relatively low^i^ | 0.70  (0.26-1.86) | .469 | 2.74  (1.55-4.84) | <.001 | 2.41  (1.43-4.07) | <.001 | 1.86  (1.07-3.22) | .027 | 0.94  (0.36-2.50) | .906 | 2.11  (0.81-5.52) | .127 |
|  | Model P value | <.001 | | <.001 | | <.001 | | <.001 | | .051 | | <.001 | |
| **Search subscale** | | | | | | | | | | | | | |
|  | Relatively low^j^ | 0.82  (0.32-2.10) | .685 | 2.66  (1.52-4.62) | <.001 | 2.02  (1.22-3.35) | .007 | 2.08  (1.12-3.86) | .020 | 0.91  (0.36-2.33) | .849 | 2.11  (0.85-5.26) | .108 |
|  | Model *P* value | <.001 | | <.001 | | <.001 | | <.001 | | .050 | | <.001 | |
| **Usage subscale** | | | | | | | | | | | | | |
|  | Relatively low^k^ | 0.72  (0.28-1.84) | .487 | 2.00  (1.18-3.37) | .010 | 2.12  (1.29-3.50) | .003 | 1.83  (1.08-3.11) | .025 | 0.83  (0.33-2.10) | .687 | 1.70  (0.71-4.06) | .235 |
|  | Model *P* value | <.001 | | <.001 | | <.001 | | <.001 | | .048 | | <.001 | |
| **Evaluation subscale** | | | | | | | | | | | | | |
|  | Relatively low^l^ | 0.56  (0.20-1.57) | .272 | 3.01  (1.63-5.55) | <.001 | 2.71  (1.54-4.76) | <.001 | 2.08  (1.07-4.06) | .031 | 1.54  (0.49-4.87) | .464 | 2.06  (1.01-4.22) | .047 |
|  | Model *P* value | <.001 | | <.001 | | <.001 | | <.001 | | .042 | | <.001 | |

^a^eHEALS: eHealth Literacy Scale.

^b^HPLP: health-promoting lifestyle profile.

^c^SA: self-actualization.

^d^HR: health responsibility.

^e^IS: interpersonal support.

^f^SM: stress management.

^g^Adjusted for sex, institution orientation, daily reading time, daily screen time, primary information channel, and perceived health status.

^h^OR: odds ratio.

^i^Compared with relatively high overall eHEALS scores.

^j^Compared with relatively high eHEALS search scores.

^k^Compared with relatively high eHEALS usage scores.

^l^Compared with relatively high eHEALS evaluation scores.
